# A Modified Dragonfly Optimization Algorithm for Single- and Multiobjective Problems Using Brownian Motion

**DOI:** 10.1155/2019/6871298

**Published:** 2019-06-02

**Authors:** Çiğdem İnan Acı, Hakan Gülcan

**Affiliations:** ^1^Mersin University, Department of Computer Engineering, Mersin 33343, Turkey; ^2^Mersin University, Department of Electrical-Electronics Engineering, Mersin 33343, Turkey

## Abstract

The dragonfly algorithm (DA) is one of the optimization techniques developed in recent years. The random flying behavior of dragonflies in nature is modeled in the DA using the Levy flight mechanism (LFM). However, LFM has disadvantages such as the overflowing of the search area and interruption of random flights due to its big searching steps. In this study, an algorithm, known as the Brownian motion, is used to improve the randomization stage of the DA. The modified DA was applied to 15 single-objective and 6 multiobjective problems and then compared with the original algorithm. The modified DA provided up to 90% improvement compared to the original algorithm's minimum point access. The modified algorithm was also applied to welded beam design, a well-known benchmark problem, and thus was able to calculate the optimum cost 20% lower.

## 1. Introduction

The fact that the real-life problems have increased from the past to the present led the scientists to produce more effective solutions by using optimization algorithms. The search for these effective solutions brought about the understanding of the behaviors of swarms in nature. Scientists have developed various algorithms by following the behavior, experiences, and reactions of swarms in nature. These algorithms are known as swarm-inspired optimization algorithms.

So far, swarm-inspired optimization algorithms have successfully solved a lot of real-world problems: In 2013, the artificial bee colony (ABC) algorithm was used for data collection in wireless sensor networks [1], and the ant colony optimization (ACO) algorithm was used for multicompartment vehicle routing problems [2]. In 2015, the ABC algorithm was used for brain tumor segmentation in MRI images [3], and the ACO algorithm was used in job scheduling [4], in economic dispatch problems [5] and for task-scheduling problems in cloud computing [6]. In 2017, the multilevel image thresholding problem was solved with the elephant herding optimization (EHO) algorithm [7], and the ACO algorithm was used in estimating transportation energy demand in Turkey [8]. The dragonfly algorithm (DA) was used in the synthesis of concentric circular antenna arrays by Babayigit [9], and the EHO algorithm was used in support vector machine parameter tuning [10]. Lastly, Debnath et al. [11] made an important study on access point planning for disaster scenarios by using the DA in 2018.

As a result of the modification of nature-inspired optimization algorithms and combining them with other optimization algorithms or methods, hybrid optimization algorithms, which provide better results than the original ones, were developed. Hybrid optimization algorithms also solved many problems in previous studies: In 2010, the ACO algorithm for solving a complex combinatorial optimization problem was modified by Yang and Zhuang [12], and the particle swarm optimization (PSO) for nonconvex economic dispatch problems was improved by Roh et al. [13]. In 2011, Yu et al. [14] improved the ACO algorithm for the multidepot vehicle routing problem, and the ACO algorithm for constrained optimization problems was modified [15]. In 2012, the ACO algorithm on real-parameter optimization was improved [16], and Ishaque et al. [17] hybridized the PSO with the maximum power point tracking method for the photovoltaic system. In 2015, Forsati et al. [18] modified the ACO algorithm for document clustering. In 2016, Salam et al. [19] proposed a hybrid DA with an extreme learning machine for prediction, and lastly, a memory-based DA for numerical optimization problems was proposed in 2017 [20].

Random motion (randomization) is one of the most fundamental features in optimization algorithms to solve problems effectively. Random mobility ensures that there is no single way to solve the problem. The solution found during optimization suggests that even if it is the closest to the optimum and even optimal, random behavior can always be a better solution. This recommendation often prevents the best solution from getting stuck in the local best of problems. Another important benefit of random motion is the success of leaving no scanned space in the search space. If there is no random action, the optimization can only be installed in one region of the designated search space and may never see the results in other regions. Random motion increases the capacity of the algorithm to reach every field in the search space.

The classical random motion which is randomization based on a random number to be generated by the processor is commonly used in optimization algorithms. However, the occasional inadequacy of this classic randomness solution has led researchers to find new solutions. One of the new solutions to hybridize optimization algorithms with a random flight method is the Levy flight mechanism (LFM). The LFM also has a random number to be generated by the processor, but the mechanism is based on a statistical mathematical formula. There are many examples in the literature using LFM for randomization: In 2007, Pavlyukevich [21] used LFM in his research to theoretically validate and justify a new stochastic algorithm for global optimization. In 2008, Barthelemy et al. [22] used the LFM to optimize transmission and transmission of light. In 2009, Yang and Deb [23] implemented the LFM into the cuckoo search algorithm which is based on the obligate brood parasitic behavior of some cuckoo species in combination with the LFM behavior. Yang [24] worked on the firefly algorithm to adapt LFM, and the numerical results of his study proved that the proposed algorithm is superior to existing metaheuristic algorithms. In 2010, Lin et al. [25] proposed a bat algorithm with LFM for parameter estimation in nonlinear dynamic biological systems. Hakli and Uğuz [26] implemented the PSO algorithm with LFM in 2014. In that study, an improvement was achieved with LFM, and successful results were obtained due to the problem of early convergence of the agents during the optimization and localization of the agents. In 2017, Heidari and Pahlavani [27] adapted LFM to the gray wolf optimization. Similarly to the problem in PSO, they predicted that the lack of location of the wolves caused local minimization and solved this problem with the LFM.

The DA developed by Mirjalili [28] with the LFM was used to model the search process for the optimal solution of dragonflies when there is no neighborhood solution. Nevertheless, the random motion of dragonflies is intermittently interrupted by LFM and the step control mechanism within the algorithm. The LFM's very large searching steps caused interruption, and dragonflies could extend beyond the search space. In order to prevent overflowing, a step control mechanism was applied to the original algorithm. However, the step control mechanism is contrary to the original movement of the dragonflies and disrupts the nature of swarm behavior.

The main objective of this study is an adaptation of the Brownian motion to DA instead of LFM and its application to benchmark functions as available in the literature [29]. The goal of our study is to improve the performance of the DA and overcome the interruption problem caused by LFM. The reason for choosing the Brownian motion method is that its isotropic approach (completely independent of direction) increases the discovery capability. On the contrary, the sizes of the steps, both controllable size and time-based random form, prevent the outgoing of the search space, providing continuity of motion. In addition, there is only one study in which the Brownian motion has been used so far in the area of optimization: An optimization method was developed by using the Brownian motion of gas molecules in nature and very successful results were obtained in the study [29]. These results were compared with those of well-known heuristic algorithms such as PSO and genetic algorithm (GA). Within the scope of Abdechiri et al.'s [29] study, there are two aims to be achieved: (i) to increase the effect of random motion in metaheuristic algorithms and (ii) to present the contribution of the Brownian motion to swarm intelligence algorithms. Given the ease of implementation and the results of the previous study, the Brownian motion has a high potential to improve the performance of swarm-inspired optimization algorithms.

In this study, the randomization stage of DA is improved by means of the Brownian motion. The modified DA was compared with the original DA and tested in the optimization of single-objective and multiobjective benchmark functions. The results obtained from single-objective optimization functions were compared to the minimum point found, and the average values were calculated from 200 separate solutions of the benchmark functions. As a result of these comparisons, 11 of 15 benchmark functions managed to find better minimum points than the original DA. In multiobjective optimization, 5 of 6 benchmark functions achieved better results than the original DA in graphical results obtained from 100 iterations. The modified DA was finally applied to the welded beam design problem, which is a well-known real-life problem in the optimization field. According to the results, the modified DA found 20% better optimal cost than the original one. The rest of the paper is organized as follows: Section 2 presents detailed information about the materials and methods used in the study, Section 3 outlines the test methods and results of the study, and Section 4 concludes the paper.

## 2. Materials and Methods

### 2.1. Dragonfly Algorithm (DA)

The DA was developed by Mirjalili at Griffith University in 2016 [28]. This technique, which is a metaheuristic algorithm based on swarm intelligence, is inspired by the static and dynamic behaviors of dragonflies in nature. There are two main stages of optimization: exploration and exploitation. These two phases were modeled by dragonflies, either dynamically or statically searching for food or avoiding the enemy.

There are two cases where swarm intelligence emerges in dragonflies: feeding and migration. Feeding is modeled as a static swarm in optimization; migration is modeled as a dynamic swarm. According to Craig and Hart [30], the swarms have three specific behaviors: separation, alignment, and cohesion. Here, the concept of separation means that an individual in the swarm avoids static collision with his neighbor (equation (1)). Alignment refers to the speed at which the agents are matched with the neighboring individuals (equation (2)). Finally, the concept of cohesion shows the tendency of individuals towards the centre of the herd (equation (3)).

Two additional behaviors are added to these three basic behaviors in DA: moving towards food and avoiding the enemy. The reason for adding these behaviors to the algorithm is that the main purpose of each swarm is to survive. Therefore, when all individuals are moving towards food sources (equation (4)), they must avoid the enemy in the same time period (equation (5)). Each of these behaviors is mathematically modeled as follows:

(1) $$\begin{array}{c} S_{i}=\;\;-\overset{N}{\underset{j=1}{\sum }}X-X_{j}, \end{array}$$

(2) $$\begin{array}{c} A_{i}=\;\;\frac{\sum_{j=1}^{N}V_{j}}{N}, \end{array}$$

(3) $$\begin{array}{c} C_{i}=\;\;\frac{\sum_{j=1}^{N}X_{j}}{N}-X, \end{array}$$

(4) $$\begin{array}{c} F_{i}=\;\;X^{+}-X, \end{array}$$

(5) $$\begin{array}{c} E_{i}=\;\;X^{-}+X. \end{array}$$

In the above equations, *X* represents the instantaneous position of the individual, while *X*_*j*_ represents the instantaneous position of the *j*^th^ individual. *N* represents the number of neighboring individuals, while *V*_*j*_ represents the speed of the *j*^th^ neighboring individual. *X*^+^ and *X*^−^ represent the location of the food source and enemy source, respectively.

In order to update the position of artificial dragonflies in the search space and simulate their motions, two vectors are considered: step (Δ*X*) and position (*X*). The step vector, which can also be considered as speed, indicates the direction of dragonfly motions (equation (6)). After calculating the step vector, the position vector is updated (equation (7)):

(6) $$\begin{array}{c} \nabla X_{t+1}=\left(sS_{i}+aA_{i}+cC_{i}+fF_{i}+eE_{i}\right)+w\nabla X_{t}, \end{array}$$

(7) $$\begin{array}{c} X_{t+1}=X_{t}+\nabla X_{t+1}, \end{array}$$

where the values of *s*, *a*, and *c* in equation (6) represent separation, alignment, and cohesion coefficients, respectively, and *f*, *e*, *w*, and *t* values represent the food factor, enemy factor, inertia coefficient, and iteration number, respectively. This coefficient and the mentioned factors enable to perform exploratory and exploitative behaviors during optimization. In the dynamic swarm, dragonflies tend to align their flight. In the static motion, the alignment is very low, while the fit to attack the enemy is very high. Therefore, the coefficient of alignment is high and the cohesion coefficient is low in the exploration process; in the exploitation process, the coefficient of alignment is low and the coefficient of cohesion is high.

### 2.2. Levy Flight Mechanism (LFM) and Dragonfly Algorithm

**Figure 1:**
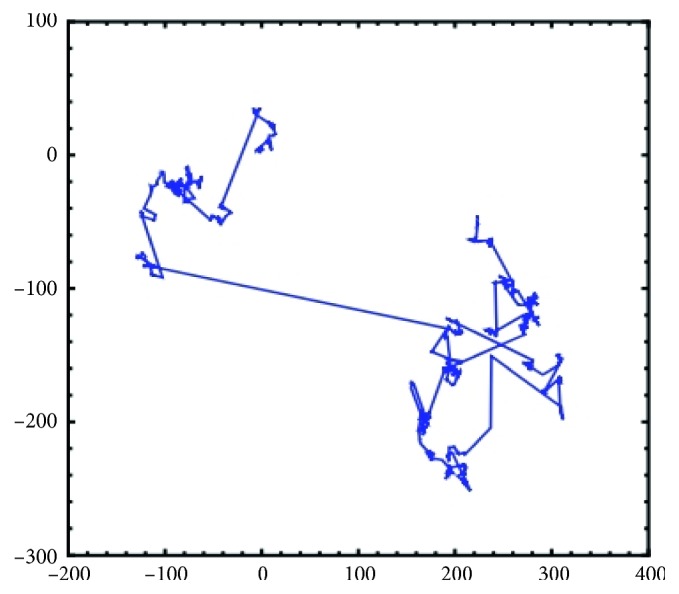
Simulation of the first 1000 steps of LFM.

LFM derives its name from the French mathematician Paul Levy. Technically, this mechanism has an infinite variance (possible length).  Figure 1 shows the simulation of LFM in the first 1000 steps.

In order to improve the randomness, the probabilistic behavior, and the discovery of artificial dragonflies, a random walk (LFM) solution is reached when there is no neighborhood solution. Accordingly, the position of artificial dragonflies is updated as follows:

(8) $$\begin{array}{c} X_{t+1}=X_{t}+\text{Levy}\left(d\right)\times X_{t}, \end{array}$$

(9) $$\begin{array}{c} \text{Levy}\left(x\right)=0.01\times \frac{r_{1}\times \sigma }{{\left|r_{2}\right|}^{1/\beta }}, \end{array}$$

(10) $$\begin{array}{c} \sigma ={\left(\frac{\tau \left(1+\beta \right)\times \sin\left(\pi \beta /2\right)}{\tau \left(\left(1+\beta \right)/2\right)\times \beta \times 2^{\left(\left(\beta -1\right)/2\right)}}\right)}^{1/\beta }, \end{array}$$

(11) $$\begin{array}{c} \tau \left(x\right)=\left(x-1\right)!, \end{array}$$

where *d* in equation (8) indicates the size of the position vector, *r*1 and *r*2 in equation (9) are random numbers in the range [0, 1], and *β* is a constant value.

In LFM used in DA, a multiplication, not included in the original mathematical formula of the flight method, was taken. This multiplication was obtained by taking 1% of LFM size as seen in equation (9). The aim here was to control the step size. This multiplication defines the value of a solution, which states the amount of the best individual deviation after LFM (position of the best individual). The 1% deviation value can be set according to the range of variables in the application. For example, if the range of variables in the application is [−10*e*6, 10*e*6], the multiplication value of 1% can be set to 1.

Although LFM raises the performance of DA to a certain extent, it is disadvantageous in that very long steps may occur depending on the characteristics of the mechanism (Figure 1). These major steps are tried to be controlled in two ways in the algorithm: The first one is that if a long step is taken, the agents have to go outside the search space and a new step vector is produced. However, it is not certain that this solution will always give correct results. The new step to be produced may take the general operation back. The second one is to take 1% of the step size as seen in equation (9) or a different percentage according to the variable range in the application. This solution method is better than the first solution. However, the step size control is contrary to the nature of LFM.

### 2.3. Brownian Motion

**Figure 2:**
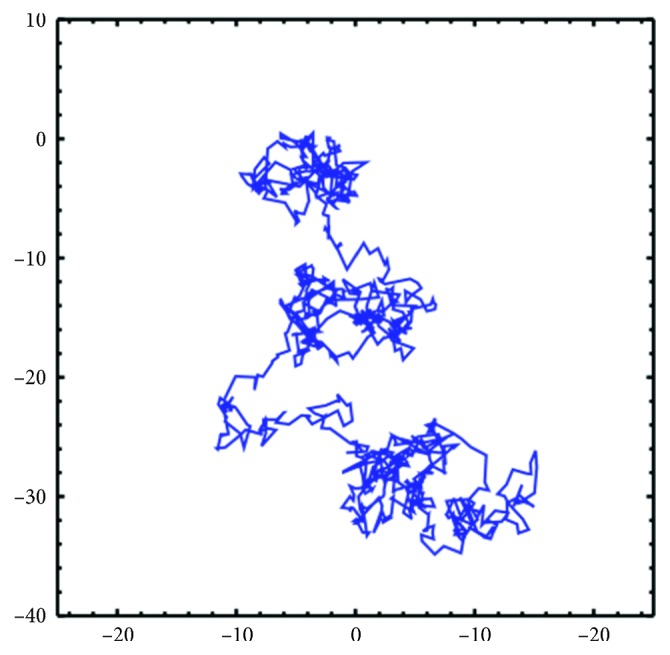
Simulation of the first 1000 steps of the Brownian motion.

Another one of the random motion mechanisms is the Brownian motion [31]. This method is inspired by the motion of free liquid/gas molecules. The introduction to the literature was done, thanks to Ingenhousz, observing the random motion of the coal and dust particles in 1779 while they were swimming in alcohol. The motion was discovered by the botanical scientist Robert Brown in 1828. The Brownian motion is defined as one of a variety of physical phenomena where a quantity continuously passes through small, random fluctuations. The Brownian motion is the random motion of particles suspended in a liquid (or a gas) resulting from collisions with fast moving molecules in the fluid. The Brownian motion is considered to be a Markov process with a Gaussian process and a continuous path that occurs continuously over time.  Figure 2 shows an example of the Brownian motion in 1000 steps.

There are some basic differences between the LFM and the Brownian motion. Mathematically, a random walk can be defined as

(12) $$\begin{array}{c} X_{N+1}=X_{N}+W_{N}, \end{array}$$

where *X*_*N*_ is the solution that exists in step *N* and *W*_*N*_ is a random vector generated from a known probability distribution. If *W*_*N*_ is produced from the Gaussian distribution, random walking is isotropic. In this case, the motion takes the form of normal diffusion and is called the Brownian motion. The expected step size can be modeled with square root scaling as follows:

(13) $$\begin{array}{c} R\left(N\right)\propto \sqrt{N}. \end{array}$$

If the steps (*W*_*N*_) are obtained from a predetermined tailed probability distribution, such as the Levy distribution or the Cauchy distribution, the diffusion becomes abnormal. In this case, the expected step size becomes

(14) $$\begin{array}{c} R\left(N\right)\propto N^{q},\;\;\;q>0. \end{array}$$

If *q* ≥ 1/2, the diffusion is called superdiffusion. Both the Levy distribution and the Cauchy distribution for step sizes may have some of the major steps leading to superdiffusion. This means that the average distance increases faster than that for normal diffusion.

### 2.4. Improvement of Dragonfly Algorithm with Brownian Motion

Modification of DA by the Brownian motion is expressed as follows:

(15) $$\begin{array}{c} X_{t+1}=X_{t}+h\;*\text{ rand}\left(\right)\;*\;P_{\text{g}}, \end{array}$$

(16) $$\begin{array}{c} h=\sqrt{\frac{T}{N}}, \end{array}$$

(17) $$\begin{array}{c} N=100\;*\;T, \end{array}$$

(18) $$\begin{array}{c} P_{\text{g}}=\frac{1}{h\sqrt{2\pi }}\exp\left(-\frac{{\left(\text{dimension}-\text{agents}\right)}^{2}}{2h^{2}}\right). \end{array}$$

In equation (16), the term *T* represents the motion time period in seconds of an agent (dragonfly). In this study, the *T* value is taken as 0.01. The term *N* in equation (17) gives the number of sudden motions (change in the path) for the same agent in proportion to time. Unlike the LFM, the Brownian motion steps are chosen based on the normal (Gaussian) distribution instead of the dominant tailed distribution. The periodic motion of the dragonflies was spread over time with a normal distribution, and sudden jumps and random motions were made in the form of vibrations. The equation was modified by the size and number of agents in the algorithm, and the Brownian motion was finalized (equation (18)).

**Figure 3:**
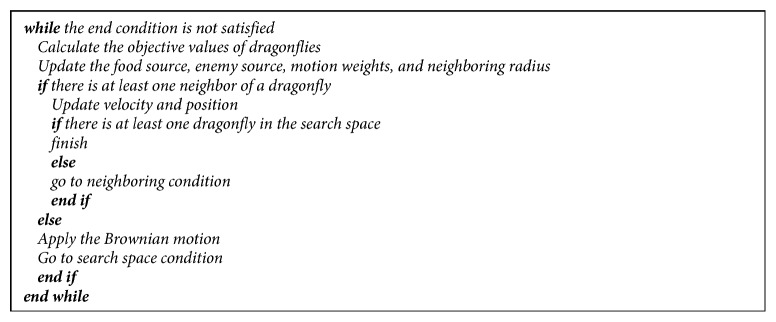
Pseudocode of the modified DA for single-objective problems.

**Figure 4:**
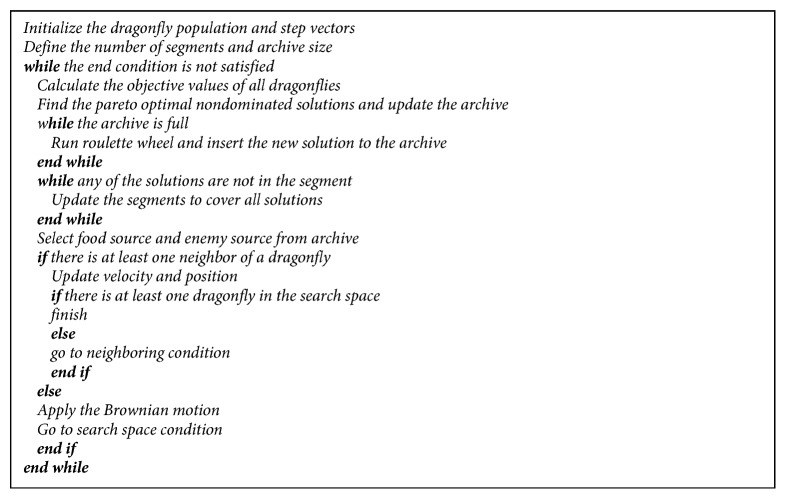
Pseudocode of the modified DA for multiobjective problems.

**Figure 5:**
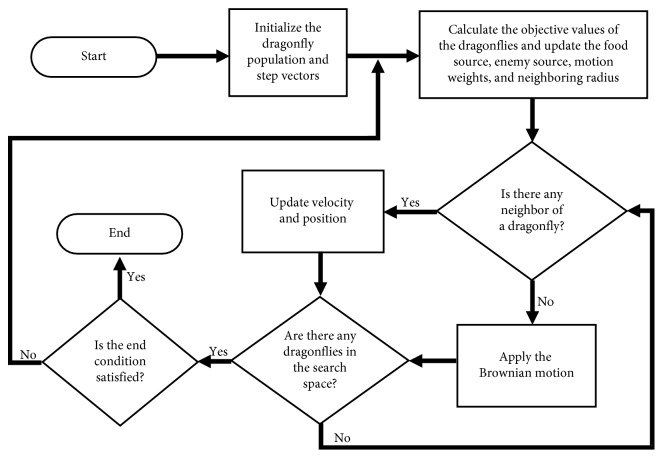
Flowchart of the modified DA for single-objective problems.

The modified DA has been adapted for both single- and multiobjective problems. Pseudocodes of the modified DA for single- and multiobjective problems are given in Figures 3 and 4.  Figure 5 shows the flowchart of the modified DA for single-objective problems. The optimization is started by randomly placing dragonflies in the search space and identifying step vectors. Then, the current position is sent as a parameter to the comparison function. After that, the best and the worst solutions are determined. Following these solutions, the number of neighborhoods for each dragonfly is examined. If each dragonfly has at least one neighbor, the velocity vector is calculated with the coefficients determined at the beginning of the algorithm and the position vector is updated. If the dragonflies have no neighborhoods, the Brownian motion solution is used and the position vector is updated accordingly. Then, it is checked whether the dragonflies are in the search space. If the control is positive, it is checked whether the criterion is fulfilled. If the control is negative, then the neighborhood is solved. Then, again, it is checked whether the criterion of completion is achieved and the optimization is terminated.

**Figure 6:**
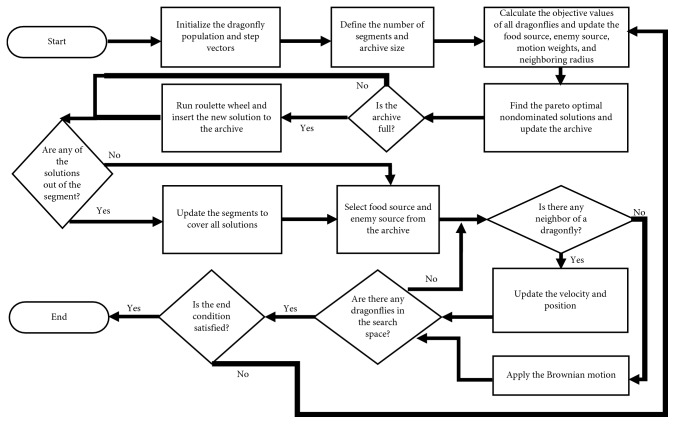
Flowchart of the modified DA for multiobjective problems.

The flowchart of the modified DA for multiobjective optimization is shown in Figure 6. Multiobjective optimization starts with placing dragonflies randomly in the search space and identifying step vectors. Then, the maximum archive size and the number of segments (hyperspheres) are defined. The instantaneous locations of the dragonflies are sent as a parameter to the comparison function, and the solutions that cannot be dominated by each other are produced. If the archive is full, some solutions are eliminated with the roulette wheel mechanism and new solutions are added to the archive. If any of the added solutions are left out of the hypersphere, the hypersphere is updated to cover all solutions. Following these processes, the best and the worst solutions in the archive are assigned as the source of nutrients and enemies, respectively. The neighborhood control is performed, and from there on, the algorithm works in the same way as the single-objective problem optimization.

## 3. Test Results and Discussions

In this section, the experimental evaluation of the modified DA is presented. The MATLAB [32] software was used for the solution of single-objective and multiobjective problem optimizations and the application of the welded beam design problem. In all cases, the computer used in simulations is configured with 2.2 GHz Intel Core i7 CPU and 6 GB DDR3 RAM. 15 benchmark functions were used for the optimization of the single-objective problems and 6 for the multiobjective problems. The results are compared and discussed with those of the original DA in terms of minimum point achieved and average performance.

### 3.1. Benchmark Functions

Test functions are useful for evaluating convergence rate, precision, robustness, and overall performance characteristics of optimization algorithms. Here, some test functions are presented to give an idea of different situations in any optimization algorithm that can be encountered when dealing with such problems. Only a general form of equality, limits of object variables, and global minimum coordinates are given here. Tables 1 and 2 show the benchmark functions used for single-objective and multiobjective problems.

### 3.2. Single-Objective Problem Optimization Results

**Figure 7:**
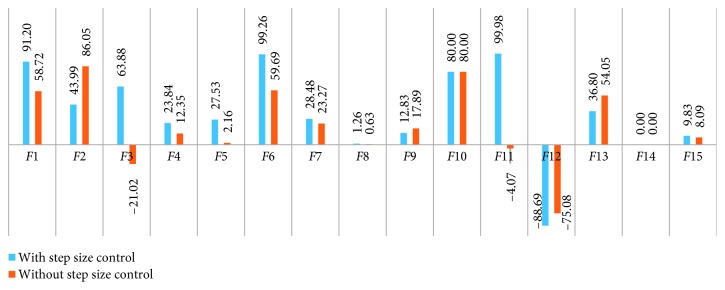
Percentage of the success rate of minimum values obtained from the modified DA.

**Figure 8:**
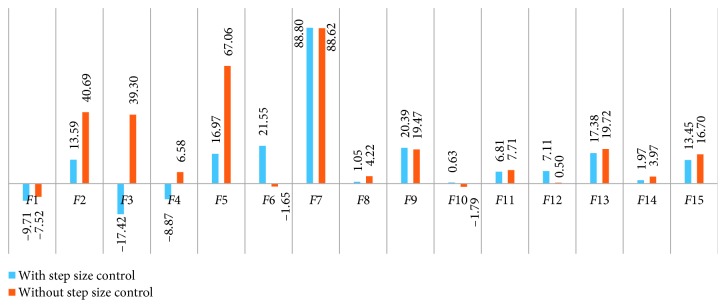
Percentage of the success rate of average values obtained from the modified DA.

The modified DA was tested by using 15 benchmark functions for single-objective problem optimization, and the minimum value for each test was compared with LFM results in the DA (the original DA). The Brownian motion in the modified algorithm was calculated separately by taking 1% of the original form and the calculated step size expressed in Section 2.3. The average value that is taken from 200 separate iterations of the original DA was compared with that of the two different versions of the modified DA: the Brownian motion and step size-controlled Brownian motion (SSCBM). The numerical results are shown in Table 3, and the graphical representation of the results is presented in Figures 7 and 8. When the results are evaluated in terms of minimum and average values, the following inferences can be made:

- In terms of minimum values, when the Brownian motion and SSCBM (the modified DA) are considered at the same time, they both gave better results than LFM (the original DA) in 11 of 15 benchmark functions. While one function did not change (*F*14) and one function reduced (*F*12), the worst (0.63%) and the best (99.98%) improvements were taken from *F*8 and *F*11, respectively.
- In terms of minimum values, the results of the Brownian motion showed performance between 0.63% and 86.05% in 11 of 15 benchmark functions. In total, three functions (*F*3, *F*11, and *F*12) failed, while there was no improvement in a function (*F*14). When two functions (*F*2 and *F*10) with the greatest success are observed, the Brownian motion is effective enough to exceed 80% success in problems with sharp boundaries. When the worst three functions are considered, the Brownian motion is not an appropriate solution for global or especially early convergent problems.
- When the results obtained from the modified DA with SSCBM were examined, they were better than those of the Brownian motion in 9 of 15 benchmark functions in terms of minimum values. SSCBM achieved over 99% success (*F*6 and *F*11) in benchmark functions that were clearly affected by step size control.
- In terms of average values, 10 of 15 benchmark functions showed better results than LFM for both versions of the modified DA. When the performance results were examined without step size control, progression was observed in 12 of 15 benchmark functions between 0.50% and 88.62%. The three functions (*F*1, *F*6, and *F*10) could not show the desired average success after 200 iterations.

(v) The biggest success was obtained from *F*7 with 88.8% in terms of average values using SSCBM. It can be said that the local minimum is quite high and SSCBM is more successful than LFM in this type of function. When the worst three functions (*F*1, *F*3, and *F*4) are examined in this manner, it can be seen that although the minimum point in global functions is more optimal than LFM, this optimality is caused by random motion because these three functions are similar and there was a decline according to the average of 200 separate results.
(vi) Finally, the Brownian motion randomization (with or without step size control), in accordance with its nature, proved its success by giving better results than LFM in most of the benchmark functions.

### 3.3. Multiobjective Problem Optimization Results

**Figure 9:**
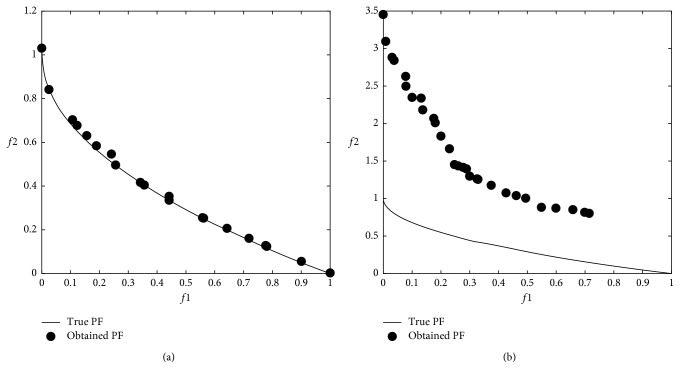
Pareto-optimal front comparison of ZDT1 optimization (PF = pareto front): (a) Brownian motion; (b) LFM.

**Figure 10:**
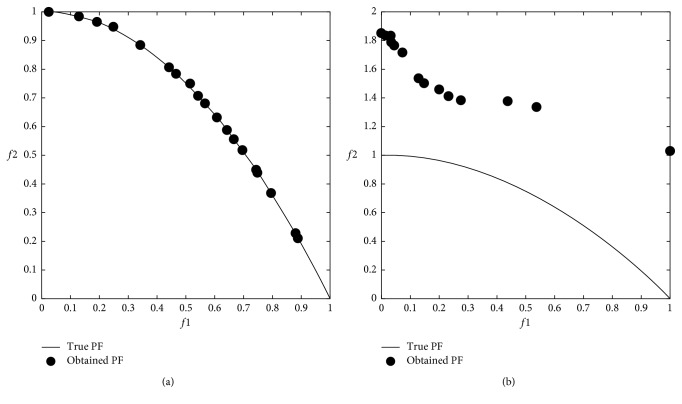
Pareto-optimal front comparison of ZDT2 optimization (PF = pareto front): (a) Brownian motion; (b) LFM.

**Figure 11:**
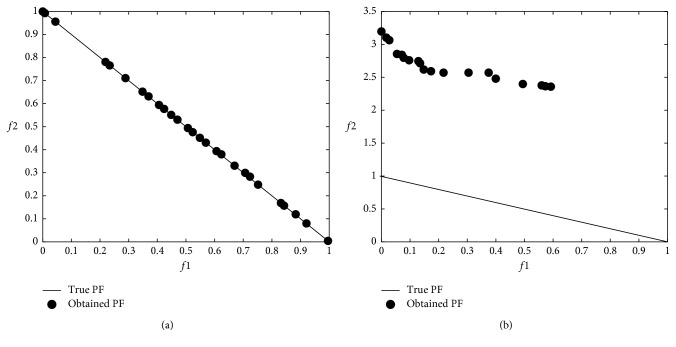
Pareto-optimal front comparison of ZDT1 linear optimization (PF = pareto front): (a) Brownian motion; (b) LFM.

**Figure 12:**
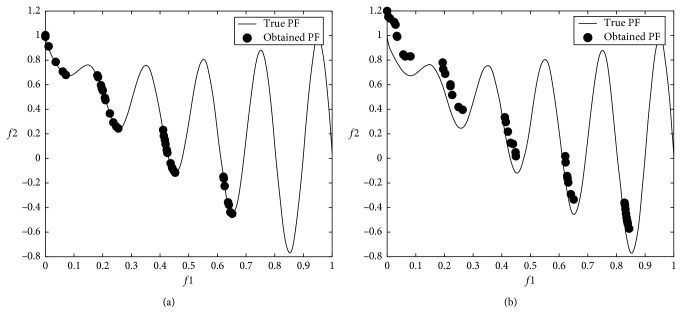
Pareto-optimal front comparison of ZDT3 optimization (PF = pareto front): (a) Brownian motion; (b) LFM.

**Figure 13:**
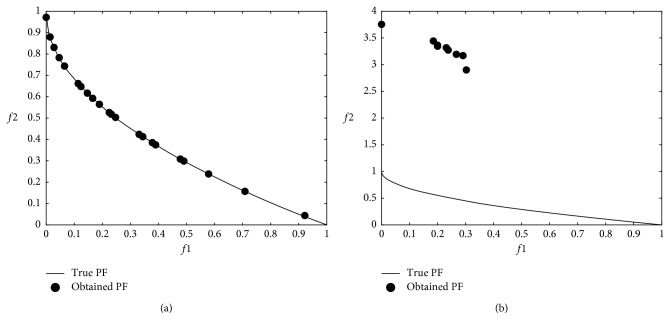
Pareto-optimal front comparison of ZDT4 optimization (PF = pareto front): (a) Brownian motion; (b) LFM.

**Figure 14:**
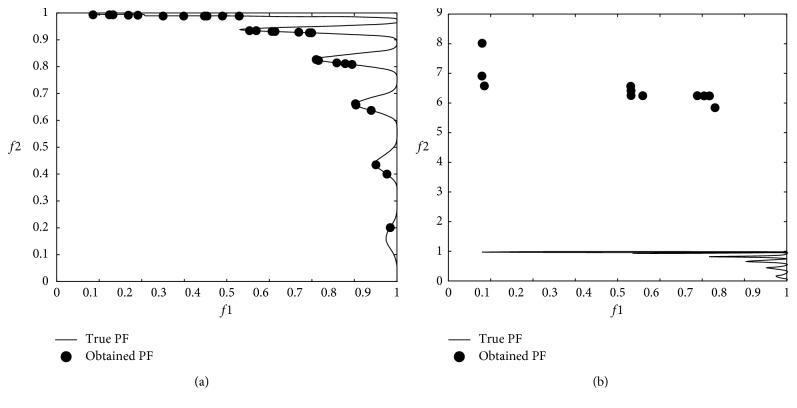
Pareto-optimal front comparison of ZDT6 optimization (PF = pareto front): (a) Brownian motion; (b) LFM.

The modified DA was applied to multiobjective problems given in Table 2, and 12 minimum values were obtained from 6 well-known benchmark functions used in the comparison. In the modified DA, the results were obtained from 100 iterations over 100 solutions which did not dominate each other after applying the Brownian motion step. The numerical *f*1 and *f*2 results are shown in Table 4, and graphical results are shown through Figures 9–14.

When the results are analyzed numerically, *f*1 values are mostly better in the original DA. This is because *f*1 minimization corresponds to the *x*1 position of artificial dragonflies in 5 of 6 problems. In the *f*2 minimization, the expected difference is observed. This is the phase where the real random motion of optimization is calculated to reach the minimum *f*2 value. Here, the Brownian motion achieved an average of 50% improvement in 5 out of 6 functions compared to LFM. If the statistical regression in a function (ZDT3) is examined, it is understood that the random motion should be applied stepwise in trigonometric rooted approaches.

When the results were analyzed graphically, the success of the Brownian motion in terms of convergence and coverage compared to LFM increased in proportion to the increase in size. While the DA with LFM was tested in 5 dimensions in the previous study [28], the search space was increased to 10 dimensions in order to increase the difficulty after it has been modified, and the Brownian motion clearly revealed its true success against the LFM.

The results of the modified DA were compared not only with those of the original algorithm but also with those of some very important optimization algorithms such as GA, PSO, and ACO by means of basic statistics (i.e., mean and standard deviation).

Table 5 shows the results of 4 algorithms on 15 different benchmark functions. These results were carried out with a total of 500 iterations with 40 agents for each optimization method.

When the results are analyzed, the proposed method for solving the benchmark functions with an early convergence problem has produced similar and successful results with those of the ACO algorithm according to other algorithms. This means that the pheromone solution used by the ants is similar to the short-step solution of the Brownian motion. The pheromone, which keeps ants close to each other and increases their nutrient concentration, has shown the effect of the neighborhood radius on the Brownian motion.

PSO was more successful than other algorithms in terms of standard deviations. The reason for this is that as in the original dragonfly algorithm, the long steps extend the search space and rarely reach the result in a shorter time. On the contrary, the proposed method has been more successful than PSO on benchmark functions with local minima. The reason for this is that the short steps to different directions in the Brownian motion method allow the particles to discover different aspects and produce better solutions at the relevant time interval.

### 3.4. A Solution of the Welded Beam Design Problem with the Modified Dragonfly Algorithm

The welded beam design is a practical design problem that is often used as a benchmark in testing different optimization techniques. The problem is an example of structural optimization problems, which consists of a nonlinear objective function and five nonlinear constraints [33]. The welded beam design problem was solved by many algorithms such as GA [34], simulated annealing [35], evolutionary strategy [36], and gravitational search algorithm [37].

In this study, the welded beam design problem was implemented in order to show the effectiveness of the modified DA. The reason for choosing this problem is that it was used many times as an application of hybrid swarm-inspired optimization techniques in the past. One of these is Kaveh and Talatahari's study [38] which hybridizes PSO and ACO. Another study is the application of the upgraded ACO [39], and Brajevic and Tuba [40] proposed a solution for limited engineering problems. Liao et al. [41] used it in the application of mixed-variable optimization problems, and finally, it is used by Ranjini and Murugan [20] for the memory-based modification of DA.

**Figure 15:**
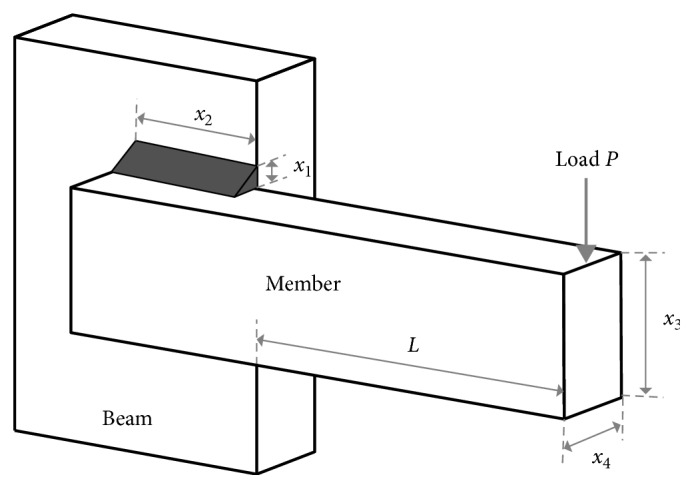
Welded beam design problem.

The welded beam design problem aims to minimize the manufacturing cost of the welded beam by finding a suitable set of four structural parameters of the beam. These four structural parameters are the thickness of the weld (*x*_1_), the length of the clamped bar (*x*_2_), the height of the bar (*x*_3_), and the thickness of the bar (*x*_4_). Relevant restrictions include shear stress (*τ*), bending stress (*θ*) in the beam, buckling load (*P*), and the last deflection of the beam (*δ*).  Figure 15 shows the systematic design of the problem.

The total cost is equal to labor costs (a function of the source dimensions) and the cost of welding and beam material. The beam will be optimized for the minimum cost by changing the source and element dimensions (*x*1, *x*2, *x*3, and *x*4). The variables *x*1 and *x*2 are usually integer multiples of 0.0625 inches but are considered to be continuous for this application. The parameters and values of the problem are given in the following equations:

(19) $$\begin{array}{c} E=30\times 10^{6}\text{ psi,} \end{array}$$

(20) $$\begin{array}{c} G=12\times 10^{6}\text{ psi,} \end{array}$$

(21) $$\begin{array}{c} L=14\text{ inches,} \end{array}$$

(22) $$\begin{array}{c} \tau_{\max}=13600\text{ psi,} \end{array}$$

(23) $$\begin{array}{c} \sigma_{\max}=30000\text{ psi}, \end{array}$$

(24) $$\begin{array}{c} \delta_{\max}=0.25\text{ inches,} \end{array}$$

(25) $$\begin{array}{c} P=6000\text{ lb}, \end{array}$$

(26) $$\begin{array}{c} f\left(x\right)=C_{0}+C_{1}+{\text{C}}_{2}. \end{array}$$

Young's modulus (psi) is given in equation (19), the shear modulus (psi) for the beam material in equation (20), the protrusion length (inches) of the member in equation (21), welding design stress (psi) in equation (22), normal design stress (psi) in equation (23) for the beam material, maximum deviation in equation (24), and load (lb) in equation (25). The cost function of the problem is shown in equation (26).

In equation (26), *C*_0 _ represents the initial cost, but it is assumed that the connections required for the installation and retention of the rod during welding are available. Therefore, the cost *C*_0_ in the total cost model can be ignored. *C*_0_ represents the cost of resources, and *C*_2_ represents the cost of materials. The welded beam design problem was carried out with 40 dragonflies with 200 iterations. The average values were obtained after the optimization was performed 200 times. The results are shown in Table 6.

According to the results obtained, although the Brownian motion was less successful in terms of the minimum cost without step size controlling, it has almost the same result with LFM. The most significant success of the Brownian motion can be seen from the average values. When no step size controlling is applied to the algorithm, the Brownian motion is more successful than LFM. This means that the long premature jumps of LFM do not always have a positive effect. The step size controlling the movement of the Brownian motion increases the likelihood of reaching optimal results. On the contrary, the Brownian motion has yielded 20% more successful results than LFM in terms of minimum cost when 1% step size controlling has been applied. The success of the modified DA will be a guide for the solution of other real-world problems according to all these results.

### 3.5. Analysis of the Modified DA

In this study, long sudden jumps which are one of the main differences between LFM and Brownian motion were examined. As previously mentioned, long sudden steps are a solution that LFM produces to avoid early convergence. However, as a result of this solution, the step produced from time to time leads to the renewal of the step when it goes out of the search space. This causes time loss. At this point, the Brownian motion solution that we have implemented rescues the algorithm from these long steps.

**Figure 16:**
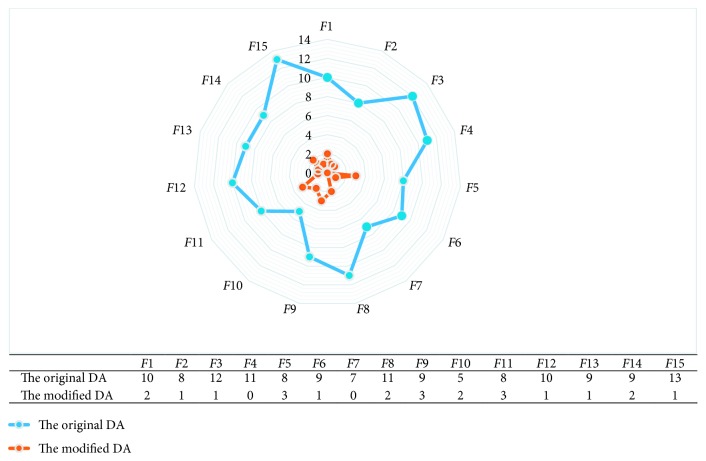
Comparison of long sudden jump counts of the original and modified algorithms.

The data obtained from Figure 16 show the long steps taken by 40 dragonflies through 1 iteration. Here, the long steps are taken as a result of the threshold value applied by taking the average distance from the steps taken for each method. As can be seen in the results, in the original DA with LFM, the long steps produced for each function are at least 5 and the average is 9.26. In the modified DA with Brownian motion, these numbers are at least 0 and average is 1.53. This is the main difference between the methods in this study, and our suggestion is that this difference often leads to success.

Even though the irregular jumps of LFM which is the motivation of the study are corrected by the Brownian motion, dragonflies may need sudden jumps to escape from the local minima. Although the proposed method greatly improves the irregular jumps caused by LFM from time to time, there is a rare possibility that it may get stuck in local minima. On the contrary, the number of neighbors in which the dragonflies have mass mobility has experienced a slight decrease with the Brownian motion. This, in a very rare way, can reduce communication groups and cause discovery to be restricted.

The modified DA has time complexity just like all other optimization algorithms. It depends on the population size and number of iterations. The overall complexity of the modified DA can be expressed as *O*(number of iterations *∗* population size). Using Brownian motion instead of LFM did not affect the time complexity of the original algorithm. The goal here is to achieve the optimum result with the optimum number of dragonflies. Apart from the number of dragonflies, the dimension of the benchmark functions and the number of iterations are among the factors affecting the execution time. However, in contrast to the number of dragonflies, the concept of dimension has an inverse proportional effect with velocity.  Table 7 compares the modified DA and the original DA in terms of execution time. The results of this comparison were obtained with 40 dragonfly agents, 15 different benchmark functions (all are in 10 dimensions), and 200 iterations.

When the results were examined, 7 of the total 15 functions improved by averagely 5%. On the contrary, there is an average of 3% regression observed for the remaining 8 functions. Two of the functions provided for improvement are outstanding: When *F*5 is examined, it has been observed that improvement in processing time is due to Brownian motion's short steps in different directions. When LFM was used, the fact that the step had to be dismissed because of the long jumps caused the execution time to be prolonged. Additionally, when *F*8 is examined from these functions, it is seen how the problem of early convergence which is the main optimization problem is solved with the Brownian motion. LFM used to solve this problem in the original algorithm does not always provide the exact solution as mentioned in the study. The Brownian motion, on the contrary, can be more successful in preventing getting stuck in local minima as it aims to go in different directions at every step. When *F*2 is examined from the observed functions, it is realized that the long steps in LFM worked this time and the short steps in the Brownian motion failed. The result to be taken as neutral here is that the success of the method may change according to the characteristic of the function.

## 4. Conclusions

Randomization is one of the essential elements of optimization techniques based on swarm intelligence. It plays a very important role in both exploration and exploitation stages. In this study, the randomization stage of DA, which is one of the swarm-based algorithms, used effectively in recent years, is modified with the Brownian motion. The results of the single-objective problem optimization from the obtained results clearly show that when the Brownian motion is used instead of LFM, definite success is achieved in the context of the minimum values in the benchmark functions. On the contrary, the modified DA was tested on 6 multiobjective problems. When numerical and graphical results are examined, it can be seen that the Brownian motion has significant success in 10-dimensional space compared to LFM.

As a general evaluation of the results, the long sudden steps from LFM sometimes caused the search space to be out of the search space. Therefore, the random motion had to be reproduced from the beginning. This is a costly time-consuming situation and it changes the original movement. However, with the Brownian motion, there is a significant reduction in the number of long steps in each iteration (Figure 16), and the original randomness is retained without having to repeat the movement. This has increased the importance of the neighborhood radius used in the original algorithm, both while saving time and facilitating the movement of dragonflies together.

In addition, the problem of getting stuck in the local minima (fast convergence) and the problem of infinite looping in the search field, as in most optimization algorithms and the original DA, have been significantly exceeded by the Brownian movement's principle of random motion generation in a different direction.

In addition to the success of LFM in relation to the usual random motion, this success of the Brownian motion also highlights the importance of random flight mechanisms. Random motion algorithms have a high effect on performance of optimization techniques. Finally, this success of the Brownian motion has shown another way to improve the results of other optimization techniques as the future work.

## Figures and Tables

**Table 1:** Benchmark functions for single-objective problem optimization.

Function	Dimension	Range
*F*1(*x*)=∑_*i*=1_^*n*^*x*_*i*_^2^	10	[−100, 100]
*F*2(*x*)=∑_*i*=1_^*n*^|*x*_*i*_|+∏_*i*=1_^*n*^|*x*_*i*_|	10	[−10, 10]
$F3\left(x\right)=\sum_{i=1}^{n}{\left(\sum_{J-1}^{\overset{˙}{I}}X_{J}\right)}^{2}$	10	[−100, 100]
*F*4(*x*)=max_*i*_{|*x*_*i*_|, 1 ≤ *i* ≤ *n*}	10	[−100, 100]
*F*5(*x*)=∑_*i*=1_^*n*−1^[100(*x*_*i*+1_ − *x*_*i*_^2^)^2^+(*x*_*i*_ − 1)^2^]	10	[−30, 30]
*F*6(*x*)=∑_*i*=1_^*n*^([*x*_*i*_+0.5])^2^	10	[−100, 100]
$F7\left(x\right)=\sum_{\overset{˙}{I}=1}^{n}ix_{i}^{4}+\text{random}\left[0,1\right)$	10	[−1.28, 1.28]
$F8\left(x\right)=\sum_{i=1}^{n}-x_{i}\sin\left(\sqrt{\left|x_{i}\right|}\right)$	10	[−500, 500]
*F*9(*x*)=∑_*i*=0_^*n*^[*x*_*i*_^2^ − 10 cos(2*πx*_*i*_)+10]	10	[−5.12, 5.12]
$F10\left(x\right)=-20\;\;\exp\left(-0.2\sqrt{\left(1/n\right)\sum_{i=1}^{n}x_{i}^{2}}\right)-\left(\exp\left(1/n\right)\sum_{i=1}^{n}\cos\left(2\pi x_{i}\right)\right)+20+e$	10	[−32, 32]
$F11\left(x\right)=\left(1/4000\right)\sum_{i=1}^{n}x_{i}^{2}-\prod_{i=1}^{n}\cos\left(\left(x_{i}/\sqrt{i}\right)+1\right)$	10	[−600, 600]
$\begin{array}{c} F12\left(x\right)=\left(\pi /n\right)\left\{10\;\sin\left(\pi y_{1}\right)+\sum_{i=1}^{n-1}{\left(y_{i}-1\right)}^{2}\left[1+10\;\sin^{2}\left(\pi y_{i+1}\right)\right]+{\left(y_{n}-1\right)}^{2}\right\}+\sum_{i=1}^{n}u\left(x_{i},10,100,4\right)\\ y_{i}=\left(\left(x_{i}+1\right)/4\right)\\ u\left(x_{i},a,k,m\right)=\;\;\left\{\begin{array}{cc} k{\left(x_{i}-a\right)}^{m} & x_{i}>a\\ 0 & -a<x_{i}<a\\ k{\left(-x_{i}-a\right)}^{m} & x_{i}<-a \end{array}\right\} \end{array}$	10	[−50, 50]
*F*13(*x*)=0.1{sin^2^(3*πx*_1_)+∑_*i*=1_^*n*^(*x*_*i*_ − 1)^2^[1+sin^2^(3*πx*_*i*_+1)]+(*x*_*n*_ − 1)^2^[1+sin^2^(2*πx*_*n*_)]}+∑_*i*=1_^*n*^*u*(*x*_*i*_, 5,100,4)	10	[−50, 50]
*F*14(*x*)=∑_*i*=1_^*n*^*x*_*i*_^2^	10	[−5, 5]
$F15\left(x\right)=\sum_{i=1}^{n}{\left(\left(x_{i}^{2}\right)/{\left(4000\right)}_{i}\right)}^{2}-\prod_{i=1}^{n}\cos\left(\left(x_{i}/\sqrt{i}\right)+1\right)$	10	[−5, 5]

**Table 2:** Benchmark functions for multiobjective problem optimization.

Problem	Definition
Zitzler–Deb–Thiele 1	Minimize *f*_1_(*x*)=*x*_1_
	Minimize *f*_2_(*x*)=*g*(*x*) *xh*(*f*_1_(*x*), *g*(*x*))
	where *g*(*x*)=1+((9/(*N* − 1))∑_*i*=2_^*N*^*x*_*i*_)
	$h\left(f_{1}\left(x\right),g\left(x\right)\right)=1-\sqrt{\left(\left(f_{1}\left(x\right)\right)/\left(g\left(x\right)\right)\right)},0\leq \;\;x_{i}\leq 1,1\leq i\leq 30$

Zitzler–Deb–Thiele 2	Minimize *f*_1_(*x*)=*x*_1_
	Minimize *f*_2_(*x*)=*g*(*x*) *xh*(*f*_1_(*x*), *g*(*x*))
	where *g*(*x*)=1+((9/(*N* − 1))∑_*i*=2_^*N*^*x*_*i*_)
	*h*(*f*_1_(*x*), *g*(*x*))=1 − ((*f*_1_(*x*))/(*g*(*x*)))^2^, 0 ≤ *x*_*i* _ ≤ 1,1 ≤ *i* ≤ 30

Zitzler–Deb–Thiele 1 (with linear pareto front)	Minimize *f*_1_(*x*)=*x*_1_
	Minimize *f*_2_(*x*)=*g*(*x*) *xh*(*f*_1_(*x*), *g*(*x*))
	where *g*(*x*)=1+(9/(*N* − 1))∑_*i*=2_^*N*^*x*_*i*_
	*h*(*f*_1_(*x*), *g*(*x*))=1 − ((*f*_1_(*x*))/(*g*(*x*))), 0 ≤ *x*_*i* _ ≤ 1,1 ≤ *i* ≤ 30

Zitzler–Deb–Thiele 3	Minimize *f*_1_(*x*)=*x*_1_
	Minimize *f*_2_(*x*)=*g*(*x*) *xh*(*f*_1_(*x*), *g*(*x*))
	where *g*(*x*)=1+(9/29)∑_*i*=2_^*N*^*x*_*i*_
	$h\left(f_{1}\left(x\right),g\left(x\right)\right)=1-\sqrt{\left(f_{1}\left(x\right)\right)/\left(g\left(x\right)\right)}-\left(\left(f_{1}\left(x\right)\right)/\left(g\left(x\right)\right)\right)\sin\left(10\pi f_{1}\left(x\right)\right),0\leq x_{i\;}\leq 1,1\leq i\leq 30$

Zitzler–Deb–Thiele 4	Minimize *f*_1_(*x*)=*x*_1_
	Minimize *f*_2_(*x*)=*g*(*x*) *xh*(*f*_1_(*x*), *g*(*x*))
	where *g*(*x*)=1+10(*N* − 1)+∑_*i*=2_^*N*^(*x*_*i*_^2^ − 10 sin(4*πx*_*i*_))
	$h\left(f_{1}\left(x\right),g\left(x\right)\right)=1-\sqrt{\left(f_{1}\left(x\right)\right)/\left(g\left(x\right)\right)},0\leq x_{i\;}\leq 1,1\leq i\leq 30$

Zitzler–Deb–Thiele 6	Minimize *f*_1_(*x*)=1 − exp(−4 *∗* *x*_1_) *∗* sin (6*πx*_1_)^6^
	Minimize *f*_2_(*x*)=*g*(*x*) *xh*(*f*_1_(*x*), *g*(*x*))
	where *g*(*x*)=1+ 9((∑_*i*=2_^*N*^*x*_*i*_)/(*N* − 1)^0.25^)
	*h*(*f*_1_(*x*), *g*(*x*))=1 − ((*f*_1_(*x*))/(*g*(*x*)))^2^, 0 ≤ *x*_*i* _ ≤ 1,1 ≤ *i* ≤ 30

**Table 3:** Comparative results of the single-objective benchmark functions.

Benchmark functions	Performance results			Percentage of success rate	
	LFM	SSCBM	Brownian motion	SSCBM	Brownian motion
*F*1 (min.)	5.56*E* − 06	4.89*E* − 07	2.29*E* − 06	91.20	58.72
*F*1 (avg.)	4.59*E* + 00	5.03*E* + 00	4.93*E* + 00	−9.71	−7.52
*F*2 (min.)	1.29*E* − 02	7.24*E* − 03	1.80*E* − 03	43.99	86.05
*F*2 (avg.)	1.46*E* + 00	1.26*E* + 00	8.68*E* − 01	13.59	40.69
*F*3 (min.)	8.39*E* − 02	3.03*E* − 02	1.02*E* − 01	63.88	−21.02
*F*3 (avg.)	1.38*E* + 02	1.63*E* + 02	8.40*E* + 01	−17.42	39.30
*F*4 (min.)	3.45*E* − 02	2.63*E* − 02	3.02*E* − 02	23.84	12.35
*F*4 (avg.)	1.91*E* + 00	2.08*E* + 00	1.78*E* + 00	−8.87	6.58
*F*5 (min.)	5.56*E* + 00	4.03*E* + 00	5.44*E* + 00	27.53	2.16
*F*5 (avg.)	1.74*E* + 03	1.45*E* + 03	5.74*E* + 02	16.97	67.06
*F*6 (min.)	4.10*E* − 07	3.04*E* − 09	1.65*E* − 07	99.26	59.69
*F*6 (avg.)	5.49*E* + 00	4.30*E* + 00	5.58*E* + 00	21.55	−1.65
*F*7 (min.)	1.41*E* − 03	1.01*E* − 03	1.09*E* − 03	28.48	23.27
*F*7 (avg.)	1.95*E* − 01	2.18*E* − 02	2.22*E* − 02	88.80	88.62
*F*8 (min.)	−3.89*E* + 03	−3.94*E* + 03	−3.92*E* + 03	1.26	0.63
*F*8 (avg.)	−2.82*E* + 03	−2.84*E* + 03	−2.93*E* + 03	1.05	4.22
*F*9 (min.)	2.99*E* + 00	2.61*E* + 00	2.46*E* + 00	12.83	17.89
*F*9 (avg.)	3.06*E* + 01	2.44*E* + 01	2.47*E* + 01	20.39	19.47
*F*10 (min.)	4.44*E* − 15	8.88*E* − 16	8.88*E* − 16	80.00	80.00
*F*10 (avg.)	2.28*E* + 00	2.26*E* + 00	2.32*E* + 00	0.63	−1.79
*F*11 (min.)	3.94*E* − 03	8.59*E* − 07	4.10*E* − 03	99.98	−4.07
*F*11 (avg.)	4.70*E* − 01	4.38*E* − 01	4.34*E* − 01	6.81	7.71
*F*12 (min.)	1.63*E* − 04	3.07*E* − 04	2.85*E* − 04	−88.69	−75.08
*F*12 (avg.)	1.29*E* + 00	1.20*E* + 00	1.29*E* + 00	7.11	0.50
*F*13 (min.)	6.70*E* − 05	4.23*E* − 05	3.08*E* − 05	36.80	54.05
*F*13 (avg.)	8.35*E* − 01	6.90*E* − 01	6.71*E* − 01	17.38	19.72
*F*14 (min.)	9.98*E* − 01	9.98*E* − 01	9.98*E* − 01	0.00	0.00
*F*14 (avg.)	1.25*E* + 00	1.23*E* + 00	1.20*E* + 00	1.97	3.97
*F*15 (min.)	3.41*E* − 04	3.08*E* − 04	3.13*E* − 04	9.83	8.09
*F*15 (avg.)	2.45*E* − 03	2.12*E* − 03	2.04*E* − 03	13.45	16.70

Note: min. = minimum; avg. = average.

**Table 4:** Results of the multiobjective benchmark functions.

Algorithm	ZDT1		ZDT2		ZDT1 linear		ZDT3		ZDT4		ZDT6	
	*f*1	*f*2	*f*1	*f*2	*f*1	*f*2	*f*1	*f*2	*f*1	*f*2	*f*1	*f*2
The original DA	0.20	2.00	0.15	1.57	0.07	1.04	0.40	0.28	0.14	2.24	0.19	1.71
The modified DA	0.21	0.50	0.50	0.72	0.55	0.45	0.22	0.43	0.20	0.60	0.61	0.91

**Table 5:** Comparison of the modified DA with well-known optimization algorithms.

Benchmark functions	Algorithms							
	The modified DA		ACO		GA		PSO	
	Mean	Std.	Mean	Std.	Mean	Std.	Mean	Std.
*F*1	4.93*E* + 00	7.16*E* − 18	5.80*E* − 19	9.85*E* − 18	1.03*E* + 03	4.47*E* + 02	5.78*E* − 18	1.80*E* − 17
*F*2	8.68*E* − 01	3.76*E* − 05	3.09*E* − 05	5.17*E* − 05	8.21*E* + 00	2.11*E* + 00	4.34*E* − 03	1.35*E* − 02
*F*3	8.40*E* + 01	2.10*E* − 06	6.04*E* − 06	2.89*E* − 06	2.68*E* + 03	1.37*E* + 03	2.60*E* − 03	4.55*E* − 03
*F*4	1.78*E* + 00	2.78*E* − 03	2.15*E* − 05	3.82*E* − 03	2.91*E* + 01	1.13*E* + 01	2.40*E* − 03	3.46*E* − 03
*F*5	5.74*E* + 02	6.79*E* + 00	7.56*E* + 00	9.33*E* + 00	1.83*E* + 05	1.17*E* + 05	8.72*E* + 01	1.10*E* + 02
*F*6	5.58*E* + 00	1.32*E* − 15	5.73*E* − 15	1.82*E* − 15	7.75*E* + 02	3.16*E* + 02	6.00*E* − 15	1.90*E* − 16
*F*7	2.22*E* − 02	4.69*E* − 03	1.42*E* − 02	6.45*E* − 03	2.29*E* − 01	9.98*E* − 02	8.21*E* − 03	4.93*E* − 03
*F*8	−2.93*E* + 03	3.84*E* + 02	−3.93*E* + 03	5.28*E* + 02	−4.68*E* + 03	1.11*E* + 02	−9.76*E* + 09	1.65*E* + 12
*F*9	2.47*E* + 01	9.48*E* + 00	2.20*E* + 01	1.30*E* + 01	3.51*E* + 01	9.17*E* + 00	1.44*E* + 01	1.08*E* + 01
*F*10	2.32*E* + 00	4.87*E* − 01	3.18*E* − 01	6.70*E* − 01	1.31*E* + 01	1.75*E* + 00	3.85*E* − 01	8.27*E* − 01
*F*11	4.34*E* − 01	7.35*E* − 02	2.66*E* − 02	1.01*E* − 01	1.06*E* + 01	4.99*E* + 00	1.15*E* − 01	4.82*E* − 02
*F*12	1.29*E* + 00	9.83*E* − 02	4.28*E* − 02	1.35*E* − 01	2.56*E* + 03	8.00*E* + 03	1.18*E* − 10	3.73*E* − 10
*F*13	6.71*E* − 01	4.63*E* − 03	3.02*E* − 03	6.37*E* − 03	9.36*E* + 04	1.21*E* + 05	3.02*E* − 03	6.37*E* − 03
*F*14	1.20*E* + 00	9.12*E* + 01	1.42*E* + 02	1.25*E* + 02	1.79*E* + 02	2.93*E* + 01	2.06*E* + 02	1.86*E* + 02
*F*15	2.04*E* − 03	8.06*E* + 01	2.67*E* + 01	1.11*E* + 02	1.60*E* + 02	2.64*E* + 01	2.59*E* + 02	2.16*E* + 02

Note: Std. = standard deviation.

**Table 6:** Comparative optimum cost results of the welded beam design problem.

Algorithm	Without SSC		10% SSC		1% SSC	
	Min.	Avg.	Min.	Avg.	Min.	Avg.
The original DA	1.302	4689.090	1.253	1.985	1.252	1.718
The modified DA	1.293	1.956	1.252	2.079	1.204	1.930

Note: SSC = step size control; Min. = minimum; Avg. = average.

**Table 7:** Comparison of execution times.

Function	Processing time (seconds)		Decrease in execution time
	The original DA	The modified DA	
*F*1	16.055	15.252	5%
*F*2	15.543	16.104	−4%
*F*3	15.643	15.742	−1%
*F*4	15.390	15.710	−2%
*F*5	17.125	15.430	10%
*F*6	14.883	15.290	−3%
*F*7	15.800	15.396	3%
*F*8	17.594	15.886	10%
*F*9	15.008	15.658	−4%
*F*10	15.145	14.758	3%
*F*11	15.307	15.034	2%
*F*12	16.439	16.000	3%
*F*13	15.389	15.994	−4%
*F*14	13.254	13.478	−2%
*F*15	14.675	15.221	−4%

## Data Availability

No data were used to support this study.
